# Effects of Long-Term Exposure to an Electronic Containment System on the Behaviour and Welfare of Domestic Cats

**DOI:** 10.1371/journal.pone.0162073

**Published:** 2016-09-07

**Authors:** Naïma Kasbaoui, Jonathan Cooper, Daniel S. Mills, Oliver Burman

**Affiliations:** Animal Behaviour, Cognition and Welfare Research Group, School of Life Sciences, University of Lincoln, Lincoln, Lincolnshire, LN6 7DL, United Kingdom; Universidade do Porto Instituto de Biologia Molecular e Celular, PORTUGAL

## Abstract

Free-roaming cats are exposed to a variety of risks, including involvement in road traffic accidents. One way of mitigating these risks is to contain cats, for example using an electronic boundary fence system that delivers an electric ‘correction’ via a collar if a cat ignores a warning cue and attempts to cross the boundary. However, concerns have been expressed over the welfare impact of such systems. Our aim was to determine if long-term exposure to an electronic containment system was associated with reduced cat welfare. We compared 46 owned domestic cats: 23 cats that had been contained by an electronic containment system for more than 12 months (AF group); and 23 cats with no containment system that were able to roam more widely (C group). We assessed the cats’ behavioural responses and welfare via four behavioural tests (unfamiliar person test; novel object test; sudden noise test; cognitive bias test) and an owner questionnaire. In the unfamiliar person test, C group lip-licked more than the AF group, whilst the AF group looked at, explored and interacted more with the unfamiliar person than C group. In the novel object test, the AF group looked at and explored the object more than C group. No significant differences were found between AF and C groups for the sudden noise or cognitive bias tests. Regarding the questionnaire, C group owners thought their cats showed more irritable behaviour and AF owners thought that their cats toileted inappropriately more often than C owners. Overall, AF cats were less neophobic than C cats and there was no evidence of significant differences between the populations in general affective state. These findings indicate that an electronic boundary fence with clear pre-warning cues does not impair the long term quality of life of cats.

## Introduction

Many domestic cats are allowed to roam freely outside of their owner’s property boundaries during the whole day or part of it. As a consequence, these cats have increased exposure to a number of risks including diseases [1], poisoning [2–4] and injury [5–6] compared to indoor cats. Although attitudes towards cat containment vary [7–8], one way of mitigating some of these risks is to constrain the cats’ free-roaming to a greater or lesser extent. Electronic containment systems potentially provide animals with more freedom than indoor housing as they allow access to an outdoor environment defined by the limits of the containment system. They have been used in a range of both pet and farm species [9]. This system relies on a wire, either buried in the ground or attached to an existing fence, which emits a low frequency radio signal. The animal wears a receiver around its neck. If the animal approaches the boundary area defined by the wire, a warning sound is emitted. If the animal continues its approach (i.e. does not remove itself from the boundary area); the collar emits an electric pulse to the skin of the animal, as a deterrent to it crossing the boundary. Although the animal may receive an electric pulse, the system is different from conventional electric fencing where it is the fence itself that is electrified. Electric fencing is widely accepted for use in various livestock species [10], but the use of collar-dependent electronic containment systems (e.g. Freedom Fence^®^, Dog Fence^®^) has raised strong opinions and concerns about their potential welfare impact. For example, in Wales, a ban on all electric aids including the virtual fencing system has been in force for several years [11].

The efficacy of virtual electronic containment system has been examined in several species including cattle [12–15] and sheep [16] and it has been found that the animals readily learn to avoid exclusion areas and associate the audio cue with the potential aversive stimulus [14]. However, the long term welfare implications of the electronic containment system have not been directly studied in any species. Exposure to virtual fences has been found to be associated initially with weight loss in cattle [13], which has been attributed to interference with grazing and/or the stress of training and testing, although after a few days cattle seemed to habituate to the system. Heifers and steers wearing these collars have also been reported to gain less weight than a control group [12], and this may have been the consequence of exclusion from riparian areas and the associated vegetation. In other studies [14, 16], behaviour measurements related to avoidance have been assessed (for example “turn”, “back up”), but no tests have been performed to specifically assess the long term welfare or affective state of the animals. The only behavioural measurements not involving avoidance were reported in a study on cattle stating that “No changes in general activity or lying behavior were found” [15], suggesting that the general behaviour of the cows was not affected during the time of the experiment.

Whilst these studies provide some information on efficacy and consequences for productivity, they do not provide adequate data for drawing firm conclusions about the welfare impact of exposure to electronic containment systems. In addition, the duration of exposure to the containment system varied in these studies from as little as a few hours [16] to, at most, two months [13]. Containment systems, particularly those used for a companion animal such as the cat, are likely to be used for several years. Being contained can restrict the cat’s ability to control its local environment by limiting its ranging area [17]. The mean daily home range of a free ranging domestic owned cat has been estimated as approximately 2ha or 20,000m2 [18, 19] whilst typically contained areas or gardens are much smaller. This may therefore reduce opportunity for exercise, exploratory behaviour and environmental interaction as well as frustrate or thwart activities that require the cat to overcome the barrier such as seeking or pursuing prey outside of the property boundaries [20].

Chronic stress could arise from receiving repeated electric corrections [21] should the cat test the boundary regularly or from the behavioural or spatial restriction of being contained, or from repeated thwarting of highly motivated behavioural responses. Chronic stress is associated with changes in affective state (i.e. emotions and moods): for example it increases anxiety in mice [22]; induces pessimistic judgement and learning deficit in sheep [23] and shifts behavioural response to a more rigid stimulus-response learning strategy in both mice and humans [24]. Chronic stress also appears to alter cognitive function, including spatial learning and memory [25] whilst chronic exposure to mild stressors induces ‘pessimistic’ judgment bias in rodents [26]. Finally, chronic stress associated with confinement has been linked to the onset of stereotypic behaviour including pacing in carnivores [27, 28]. If electronic containment represents a chronic or severe stressor, then it would be anticipated that similar changes would occur in cats contained in this way.

Therefore the aim of this study was to investigate the effect of long-term exposure to an invisible or virtual electronic containment system on the behaviour and welfare of cats. We were particularly interested in measures related to their affective state. We hypothesised that reduced interaction with an unfamiliar person, reduced exploration and interaction with a novel object, heightened sensitivity to sudden noise, and a ‘pessimistic’ judgement would indicate compromised welfare, and that deterioration in welfare would also be noted in owner reports of their pet’s behaviour [29].

## Materials and Methods

### Ethics statement

The experimental protocol was approved by the University of Lincoln Ethics Committee. Data was collected from homes in England where virtual fences are legal means for containing cats at the time of study (2014) and installed by accredited suppliers at least one year prior to the study.

### Containment system tested in this study

The containment system tested in the study was the system sold by Freedom Fence^®^. It consists of a transmitter (model FF1010) sold with a boundary kit of 100m of RF emitting wire and training flags. The transmitter emits a low power, low frequency radio signal that is picked up by a receiver worn by the animal. The animal receiver was mounted on a water resistant collar; model ProLite^™^, with a safety time-out after 20 seconds (i.e. if the cat is stuck in the “exposure” zone the collar stops the electric stimuli after 20 seconds). Each collar weighed approximately 40g. Each owner received a training manual accompanying the containment system and assistance from the company (if required) for the training of their cats. Recommendations were that the cats experienced the electric stimulus at least once during training.

### Subjects: recruitment process

Subjects were cats volunteered by their owners. Two groups of cats were defined: a group of cats that had experienced the electronic containment system for more than one year, (‘Already have a Fence’ or AF group), and a group of control cats that had access outside (i.e. being allowed outside at least one hour per day) and with no specific containment system in place (‘Control’ C group). The AF group was recruited through contact with companies that supply the Freedom Fence^®^ system. The C group was recruited via a press release advertised in the Lincolnshire and Derbyshire press, and via online on cat-related websites.

Inclusion and exclusion criteria were set to include neutered cats of either gender from any breed or type, aged between one to fifteen years, who appeared in good physical health according to their owners and not receiving any long-term health or behaviour treatment. Cat health status was confirmed by the research team that included qualified veterinarians. Lactating females and those with kittens less than two months old were excluded. Also excluded were households that had gone through a change in lifestyle in the three months before the start of the study, e.g. moving house, moving a lot of furniture, having work done in the house (like building a conservatory, redecorating, painting rooms), changes in the household like arrival of a new baby or a new person moving in. This is because such disruption can influence the cat’s behaviour and affective state [30–32]. Cats included in the study could come from the same household (i.e. multi-cat households), as long as they met the inclusion criteria. Cats included in the study each underwent four behavioural tests, and their owners completed one questionnaire per cat (see S1 File).

### Behavioural tests

Long term environmental challenges such as repeated exposure to noxious stimuli and restriction of roaming can affect an animal’s perception of and responses to environmental change, so behavioural tests were chosen that focused on detecting a change in the cat’s reaction to different types of novelty (unfamiliar person, novel object, unexpected sound) and measures of affective state (cognitive bias test). The tests were carried out in a specific order on two days: unfamiliar person test, novel object test, sudden noise test on the first day. Then the cognitive judgement bias test was carried out on the second day. The tests were always carried out over two days for each household irrespective of the number of cats tested. The unfamiliar person test was carried out first as the experimenter had to be unfamiliar. Then the novel object test and the sudden noise tests were performed on the same day because they do not last long and do not necessitate training. The sudden noise test was specifically carried out last on the first day as it involved the provision of food which could influence the cat’s behaviour, and to take into account that exposure to the sudden noise may startle the cat. The cognitive judgment bias test was carried out on the second day because it necessitated training and the provision of food rewards and interaction with the experimenter, which was to be kept at a minimum for the three other tests. All behavioural tests were video recorded for analysis at a later date. Each cat was tested individually, including those coming from multi-cat households.

#### Unfamiliar person test

An animal’s response to the approach or presence of a stranger or an unfamiliar person has been evaluated using a variety of tests [33–37]. Interaction between a cat and human seems to increase with time spent in the home by a familiar human [37], the activity of the human and whether the person is familiar or not [36]. We designed the unfamiliar person test using a combination of the tests used in previous studies [33–37]. We hypothesized that cats in a negative affective state would be more cautious around an unfamiliar person due to the potential threat posed by the social novelty. We focused on how the cat reacted to an unfamiliar person when it was alone with them, whether the cat’s reaction changed depending on the person’s activity, and the effect of the owner’s presence. Our study took place in the home setting of the cat, since the focus was on the cat’s response to the presence of an unfamiliar person rather than to its general surroundings. The test consisted of four phases in the same order lasting up to two minutes per phase. The test took place in a room familiar to the cat, where it was used to having the door closed. If the cat was not used to being in the room with the door closed, a pre-training phase was initiated for two weeks before the test was performed. During this time the owner would occasionally close the door for short periods of time until the cat did not react to the door being closed (e.g. standing in front of the door or vocalising in front of the door). The same person (NK) was used as an unfamiliar person for all the cats. The test protocol is presented in Table 1.

**Table 1:** Experimental Procedure of the Unfamiliar Person Test.

Phase	Time	Description
Phase one	Up to two minutes (maximum)	The owner gently introduced the cat into the room by putting the cat on the floor just inside the entrance and closing the door behind it. The unfamiliar person sat at more than one metre from the door, hands on her knees, with no direct eye contact with the cat, ignoring the cat until it approached. For all four phases the cat was considered to have approached once it was at less than half a cat’s length from the unfamiliar person. The next phase of the test began as soon as the cat had approached the unfamiliar person or once two minutes had elapsed if no approach occurred.
Phase two	Two minutes	A: If the cat had not approached in phase one, then the unfamiliar person extended her hand and called the cat “hello X (cat’s name), come here” every 30 seconds until the cat approached. If the cat still did not approach, the cut-off point was again two minutes and the test moved on to phase three (i.e. the introduction of the owner). If the cat started to interact by looking directly at the person while close and rubbing on the person, the unfamiliar person presented her hand to rub on and stroked the cat, saying “good boy/girl” in a gentle voice. B: If the cat had already approached, the unfamiliar person presented her hand to rub on, stroking the cat if the cat initiated the interaction, saying “good boy/girl” in a gentle way. The words “hello X, come here” were repeated every 30 seconds if the cat was not in contact with the unfamiliar person. This lasted for two minutes. The unfamiliar person only interacted with the cat if the cat initiated the interaction, for example, by offering its head or back to stroke, by rubbing on the unfamiliar person, kneading, etc…Each interaction was kept very short, with the unfamiliar person pausing every other second to ensure that the cat still wanted the interaction to continue, by displaying the same initiating behaviours as previously described.
Phase three	Up to two minutes (maximum)	At the start of this stage the experimenter signalled the owner to come in by saying “you can come in”, and the owner came into the room, sat in a pre-determined place at least two metres away from the unfamiliar person, perpendicular to the unfamiliar person, with their hands on his/her knees. The unfamiliar person had the same posture. The owner was instructed to ignore their cat and make no eye contact with it during the two remaining stages. The unfamiliar person also made no direct eye contact with the cat, ignoring the cat until it approached. The next phase of the test began as soon as the cat had approached the unfamiliar person or once two minutes had elapsed if no approach occurred.
Phase four	Two minutes	A: If the cat had not approached in phase three, then the unfamiliar person extended her hand and called the cat “hello X (cat’s name), come here” every 30 seconds until the cat approached. If the cat still did not approach, the cut-off point was again two minutes. If the cat started to interact by looking directly at the person while close and rubbing on the person, the unfamiliar person presented her hand to rub on and stroked the cat, saying “good boy/girl” in a gentle voice. B: If the cat had already approached, the unfamiliar person presented her hand to rub on, stroking the cat if the cat initiated the interaction, saying “good boy/girl” in a gentle way. The words “hello X, come here” were repeated every 30 seconds if the cat was not in contact with the unfamiliar person. This lasted for two minutes.

#### Novel object test

The novel object test is used to detect emotional changes that may be related to stressful situations, with changes in alertness and exploration being useful measures and more anxious animals tending to show less interaction with novel objects [38, 39]. The test took place in the same room as for the unfamiliar person test. One of three different novel objects was used so that cats living in the same household had no chance of encountering the same object during their test. The objects were selected to be safe for the cat, suitable for a home environment, easy to clean, and unlikely to have been previously encountered by the cats. The three objects were: a small marble elephant glued on a 10cm*10cm mirror, a small obelisk glued on a 10cm*10cm mirror and a resin giraffe glued on a 10cm*10cm mirror. The addition of the mirror was intended to increase the visual novelty of the object to ensure that it was an object that the cat had never encountered before. The object was placed 1.5m away from the entrance of the room. The owner gently introduced the cat into the room and closed the door, staying outside of the room. The cat (and its potential interaction with the object) was filmed by the experimenter for three minutes from the moment the cat was introduced in the room. The use of the experimenter to film rather than an unmanned camcorder and tripod allowed the cat’s behaviour to be more fully tracked.

#### Sudden noise test

The noise test is a form of mild startle test, studying an animal’s reaction to a sudden event (visual or auditory event) when the animal is engaged in an activity such as eating. Such tests are used in a variety of species mostly to investigate fearfulness and emotional reactivity [40–42]. In our study, the test was designed to determine both the cat’s reaction to a sudden or unexpected noise while it was eating as a means of assessing anxiety and a potential stimulus generalisation. The choice of the noise was based on consideration of the following: the noise had to have no social significance (e.g. similar to a cat hissing or a dog barking or a human yelling); trigger a reaction without being excessively threatening and have a high pitch as the warning sound in the containment system is a high pitch sound. After testing a variety of potential sounds during a pilot study, the grating sound of a metallic doorway was chosen: a novel high pitch noise that lasted two seconds (average volume 76.26 ± 2.22 Db).

Two speakers were placed 30cm on one side of the cat’s normal food bowl, with the side chosen balanced across cats. The speakers were linked to a computer that was set up as far away as possible within the test room from the food bowl. The cat was present during the setting up of the apparatus and was given two minutes for habituation to the changes (i.e. presence of the speakers) near to their food bowl. The cat’s favourite food was then put into the food bowl (at least enough so that the cat could not finish the food prior to the end of the test). When the cat had been eating continuously for at least five seconds, the sudden noise was played. The cat’s behaviour was video recorded by the experimenter from the start of the test (i.e. prior to food delivery) until 50s after the playback of the sudden noise, at which point the test ended. The experimenter stood well back from the experimental setting (over two metres) and ignored the cat during the test.

#### Cognitive judgement bias test

**Fig 1:**
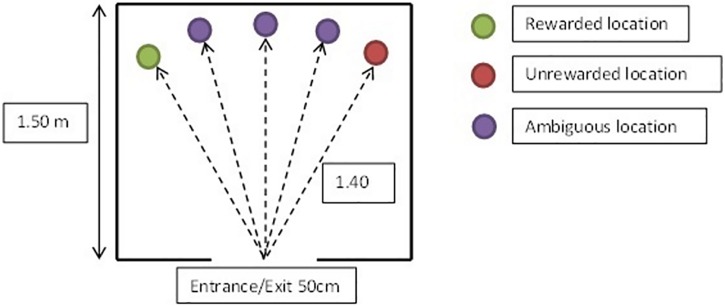
Judgment bias test arena and example of locations of the food bowl.

The cognitive judgment bias test is an established procedure in several species [43, 44], with one study establishing its potential use in cats [45]. Due to the constraint of the home setting and having to perform the training and testing in one day, we adapted a test procedure developed in dogs [46] in order to determine differences in ‘optimistic’ and ‘pessimistic’ judgment biases between the study populations. The test took place in a room familiar to the individual cat being tested. A wire mesh arena 1.5m x 1.5m and 0.25m high was used to define the training and test area (Fig 1). The cat was trained to discriminate between a rewarded location (R) and an unrewarded location (U). At the rewarded location the cat’s favourite food was available in the uppermost bowl composed of two feeding bowls stacked together. This raised bowl was used so that the presence or absence of food could not be judged from visual cues at the start point, and consequently the cats should be largely reliant on learnt spatial cues. At the unrewarded location, the food was present, between the two stacked bowls, visible through holes in the top bowl but not accessible, in order to control for olfactory cues. Whatever the location, the bowl was put at 1.4m from the entrance of the arena and the latency to get within 10cm from the bowl with the head directed towards the bowl was recorded, because at this distance the cat was able to see whether or not food was accessible in the bowl (see Fig 1).

Given previous published issues with the length of time to complete the training for cats in this sort of test [45], the motivation for the cats to complete the test was expected to be a potential challenge. The owners were therefore asked not to feed their cat for at least three hours before the beginning of the test. After a pilot study with four cats, the training and testing phase were carefully designed to maintain motivation. The training procedure was divided into sessions of six trials each, and the sessions were performed one after the other with up to 30 second breaks between each session, the pilot study having showed that longer breaks resulted in a decrease in the cat’s performance. The number of sessions depended on the individual cat’s performance, with the training phase stopping as soon as each cat had either reached the success criterion or refused to continue the test. For each trial, the cat was gently placed near the arena entrance by the researcher, who put the bowl at the predetermined location, then timed the latency to approach the bowl with a stopwatch. If the cat failed to go to the location within 15 seconds, then that particular trial was ended. The first session of the training phase always began with at least three R trials. For example, the first session could be RRRURR or RRRUUR or RRRURU or RRRUUU. During the whole training phase, no more than three U trials were given in succession. The success criterion was that for six consecutive trials involving three R and three U trials, the cat would consistently run faster to the R location than to the U location [46]. For half the cats the rewarded location was on the left side, and for the other half the rewarded location was on the right side. The cats were randomly allocated to one side (left or right for the rewarded location) within the constraint of counterbalancing the numbers in each group. The testing phase exposed the cat to three ambiguous locations of the food bowl: the near rewarded location (NR), the middle location (M) and the near unrewarded location (NU). The test consisted of four to seven trials (depending on the cat’s performance), where the ambiguous locations were presented in a pseudo-random order interspersed with rewarded or unrewarded locations. In order to maintain motivation, the testing phase began immediately after the cat reached the training criterion [46] and contained more R trials than U trials, e.g. NR R M R NU U. The ambiguous locations were presented only once to avoid potential problems due to immediate repeated testing [47].

### Owner questionnaire

Questionnaires and surveys are widely used in research to gather information about a person’s perception of a specific issue related to animals [7, 48]. An animal’s owner, specifically when the animal is a pet, is likely to be the person who spends most time with the animal and interacting with it. Owner’s perceptions are thus a useful complementary tool to behavioural testing [29]. For instance, the experience of cat owners means they can classify cat vocalisations better than non-cat owners [49], especially when it is their own cat [50].

**Fig 2:**
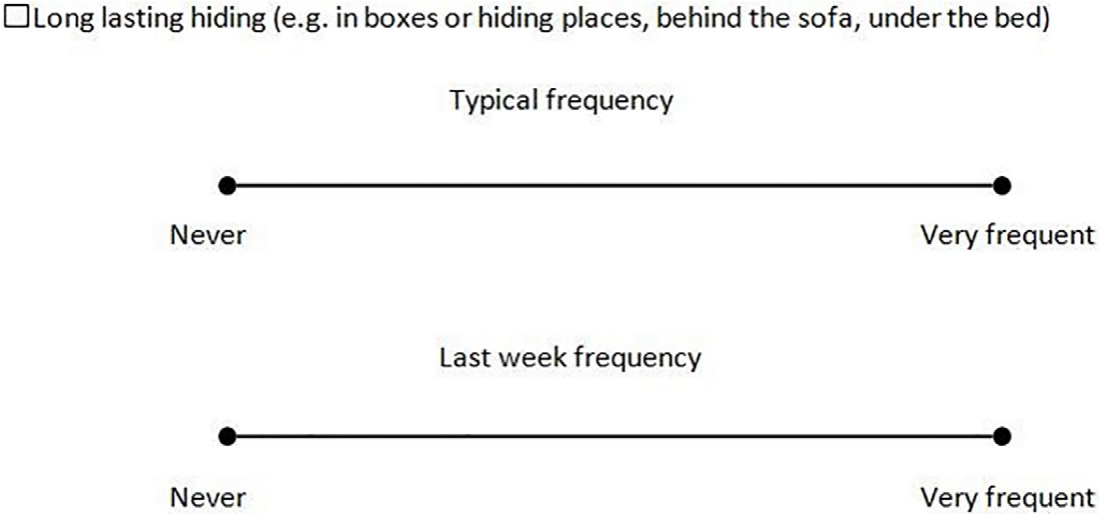
Examples of questionnaire’s items, specifically behavioural rating using a Visual Analog Scale from 0 to 9.

The questionnaire was designed to achieve three goals: to gather information about owner’s perception of their cat’s behaviour; to compare those perceptions to the behavioural tests; and to gather information regarding other potential behavioural changes in the cat. The questionnaire contained demographic items, items on outdoor access, and items about anxiety, stress and the occurrence of specific behaviours (see S1 File). The items relating to specific behaviours were rated using a single measure horizontal visual analogue scale, from 0 to 9 cm [51]. The owner was asked to rate the typical response for each behaviour and response in the last week, by marking a cross on the scale–See Fig 2.

The questionnaires were adapted for each group: for example, items specific to the containment system were removed for the control group. The questionnaires were filled in by the owner after each batch of behavioural tests. The researcher was available to answer any questions and provide terminological clarification when needed.

### Behavioural analysis

Video recordings from the Unfamiliar Person Test, the Novel Object test and the Sudden Noise Test were analysed according to the following procedure. First, a random sample of 10% of the videos was watched in order to identify the range of behaviours that were likely to be present. Then a specific ethogram was designed for each test (See S2 File), with the behavioural definitions adapted from a published ethogram for the felidae [52]. The ethograms categorized the location, locomotion, posture behaviours, and the behaviours specific to the test (i.e. interaction with the object, interaction with the unfamiliar person, and interaction with the owner). In addition, lip-licking, yawning and excessive self-grooming have been associated with anxiety and stress in the cat [53, 54]. Excessive self-grooming is described in cases of separation anxiety in the cat [55]. Such behaviours have been suggested to indicate heightened arousal or a negative affective state (e.g. anxiety, or some other form of distress such as being conflicted by a stressful situation), so were also recorded; as well as any kind of vocalisation the cat produce (e.g. meowing, purring, hissing). In case of self-grooming, since it was not possible to discriminate between normal and excessive self-grooming during the test, we recorded all grooming behaviour as a single variable.

The videos were coded with Noldus Observer 10.5, and then the data were exported and entered for analysis. The videos were coded with continuous recording, and yielded frequencies, durations and latencies of state behaviours, and frequencies of point behaviours.

### Data analysis

All behaviours performed by less than 20% of the cats were removed from analysis. Due to the high number of remaining different behaviours to analyse, data reduction was performed using a factor analysis (FA). Even with a small sample size (below 50 subjects), FA is possible provided that the variables are well correlated and that sample adequacy is achieved [56]. In order to achieve sample adequacy, a Kaiser-Meyer-Olkin measure of sample adequacy (KMO; [57]) was used, overall and on individual variables, with 0.6 taken as the absolute minimum value for the overall KMO measure, and 0.5 as an absolute minimum for individual KMO measure [58]. Bartlett’s test of sphericity was used to assess the suitability of the data to perform the analysis. Analysis was performed only when the Bartlett’s test of sphericity result was significant (p<0.05). Factors were retained as a “factor of interest” when reaching at least two of the three following criteria: an Eigen value > 0.9; explained 10% or more of the variance; visually before or included in the inflexion point of the scree plot. The variables retained to explain the factor were the ones which loaded ≥ 0.5 on the factor, positively or negatively. When FA was possible (according to the KMO measures), this was performed with an orthogonal rotation (varimax; [58]). All data were tested for normality using the Shapiro-Wilk test with p value set at 0.05. When p ≤ 0.05, the data was not distributed normally and a non-parametric Mann Whitney U test was performed. When p ≥ 0.05, the data was distributed normally and an independent t test was performed. When it was not possible (i.e. sample adequacy was not achieved) or when sample adequacy was achieved with a smaller number of behaviours than initially retained, individual behaviours of interest were tested for normality and analysed as previously stated depending on the normality test results. All statistical analyses were performed using SPSS version 22. Data is reported as mean ± SE for data analysed using parametric tests and median ± interquartile range for data analysed using non-parametric tests.

Specifically for the unfamiliar person test, due to the circumstances of the test (i.e. a field study carried out in the home environment with fixed camcorders), cats were not always in the camera’s field of view. In order, therefore, to compare durations and frequencies of behaviours between cats, all variable values were divided either: by the time that the cat was visible (for the cat body); by the time the gaze was visible (for “gaze behaviours”); and by the time the tail was visible (for “tail behaviours”). Vocalisations were divided by the test phase duration. This yielded values that were a proportion of behaviour per second, therefore comparable between cats and between groups. Each analysis was performed phase by phase.

## Results

### Demographics

Twenty three cats were recruited in each group, with 10 females and 13 males, all neutered, in each group. The AF group came from 13 separate households, with eight multi-cat households (two households with three cats, six households with two cats), age ranging from 1 to 15 years old. Each cat in the multi-cat households was equipped with a collar for the containment system. The C group came from 14 separate households with seven multi-cat households (two households with three cats, five households with two cats), with ages ranging from 2 to 13 years old. Most of the AF owners in the study (all but one) stated that they had installed the containment system to keep their cat safe from the risk of road traffic accidents. The size of the contained area ranged from 120m^2^ to 28000m^2^ (median 1821 m^2^). Most of the cats in the two groups were neutered at around six months of age, with the exception of cats adopted as adults (four cats).

### Unfamiliar person test

Twenty cats in the AF group and 19 cats in the C group provided data for analysis, seven cats being excluded because of suboptimal test conditions, for example the cat escaping from the room. In phase one (unfamiliar person alone with cat, person passive) the C group “lip licked” significantly more than the AF group (mean licks per second ± SE AF: 0.0 ± 0.0; C: 0.0048 ± 0.0383; Mann-Whitney U test U = 219, Z = 2.732, p = 0.029). There was no significant difference between the groups for the behaviours: “gaze towards stranger” duration and frequency; “tail up” duration and “head shaking” frequency (all p > 0.05).

**Fig 3:**
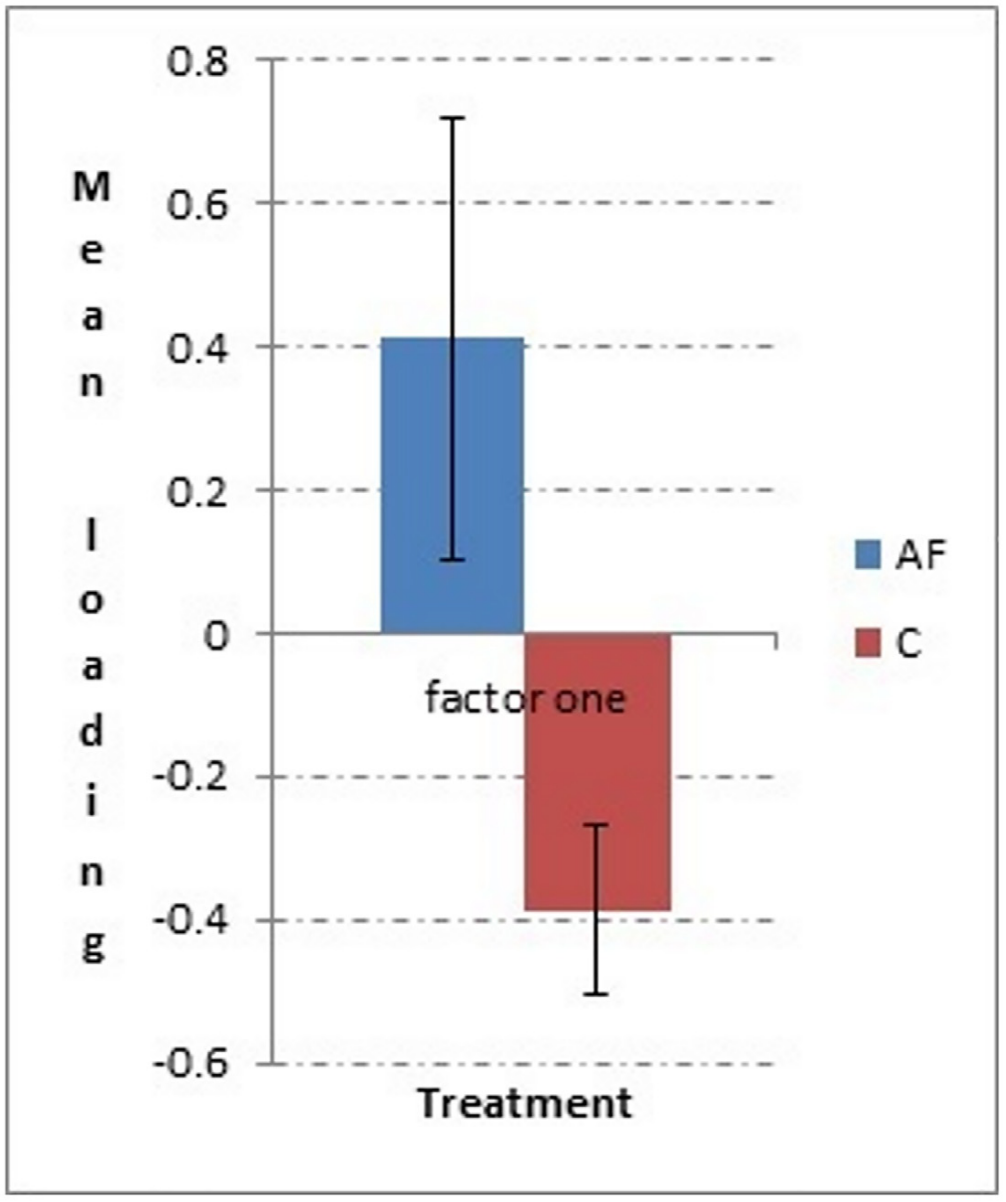
Mean ± standard error for loading on factor one “looking at and exploring the stranger” for phase two, AF group and C group.

In phase two (see S3 File), two factors were extracted during the factor analysis, which explained 64.7% of the variance. Factor one was labelled as “Looking at and exploring the stranger”, and factor two “Confidence” as the greeting behaviour loaded positively on the factor while anxiety/and conflict related behaviours loaded negatively. There was a significant difference between AF (mean loading ± SE; 0.413 ± 0.309) and C group (mean loading ± SE; -0.387 ± 0.119) for factor one “looking at and exploring the stranger” t_18.033_ = 2.335; p = 0.031 (Fig 3). There was no significant difference between AF and C group for factor two “Confidence” (p > 0.05).

In phase three (see S3 File), two factors were extracted during the factor analysis, which explained 60.4% of the variance. Factor one was named “looking at owner and greeting behaviour”, and factor two “looking at stranger and positive behaviour” as “gaze towards stranger” loaded positively on this factor while the anxiety/conflict like behaviours loaded negatively. There were no significant differences between AF and C groups for either factor (Mann Whitney U test, p > 0.05).

**Fig 4:**
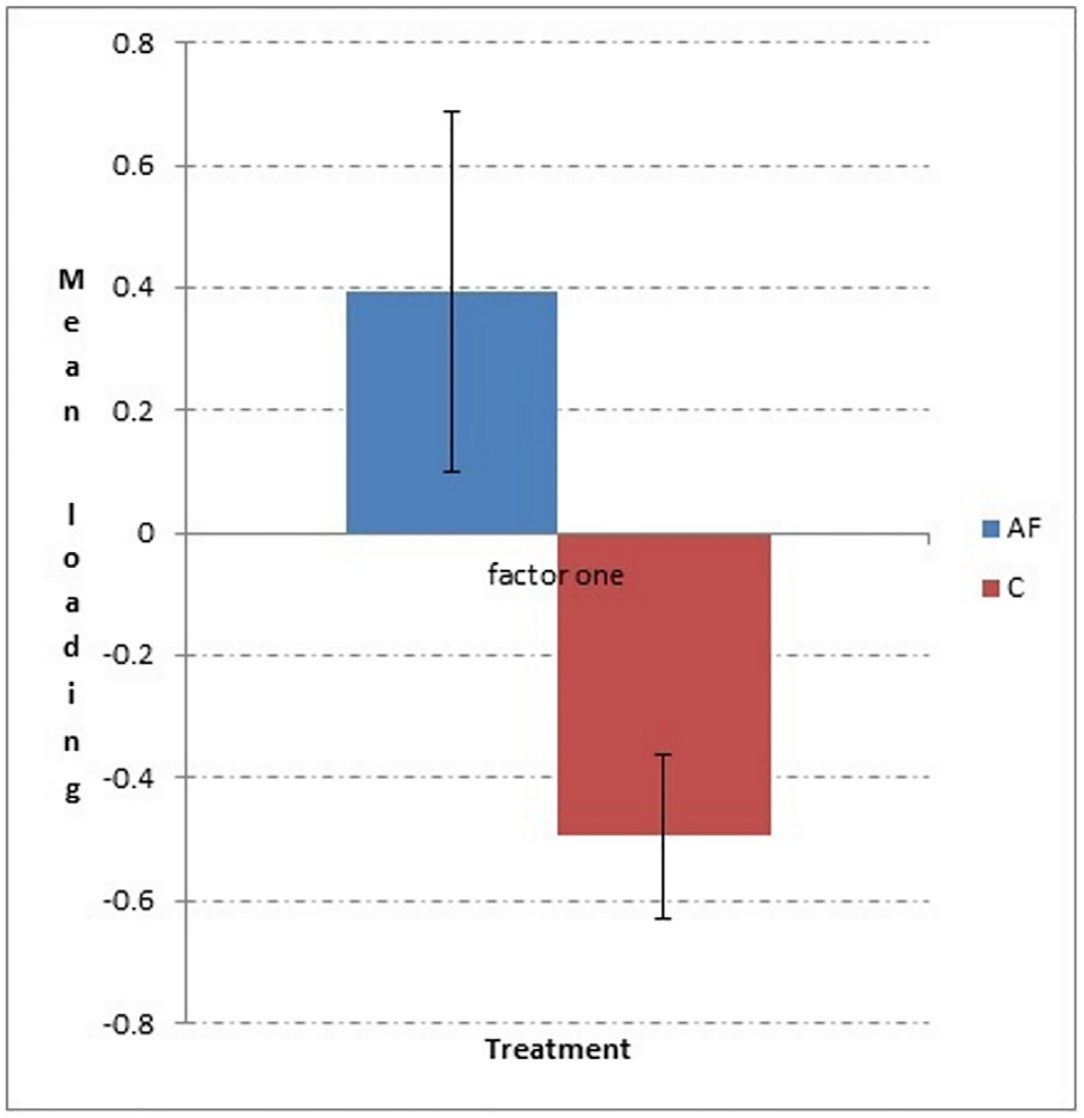
Mean ± standard error for loading on factor one for phase two, AF group and C group.

In phase four (see S3 File), two factors were extracted during the factor analysis, which explained 60.8% of the variance. Factor one was named “interaction with stranger”, and factor two “gazes and positive behaviour” as the greeting behaviour loaded positively on this factor while the anxiety/conflict like behaviours loaded negatively. There was a significant difference between the AF (mean loading ± SE; 0.396 ± 0.294) and C groups (mean loading ± SE; -0.495 ± 0.135) for factor one “interaction with stranger” t_19.859_ = 2.734; p = 0.013, with AF cats scoring, on average, higher for this factor (Fig 4). There was no significant difference (p > 0.05) between AF and C groups for factor two “gazes and positive behaviour”. Regarding individual behaviours of interest, anxiety/conflict like behaviours were selected for analysis (i.e. “lip licking” and “self-grooming” duration). There were no significant differences either for “lip licking” or “self-grooming” duration between AF and C groups (Mann Whitney U test; p > 0.05).

### Novel object test

**Fig 5:**
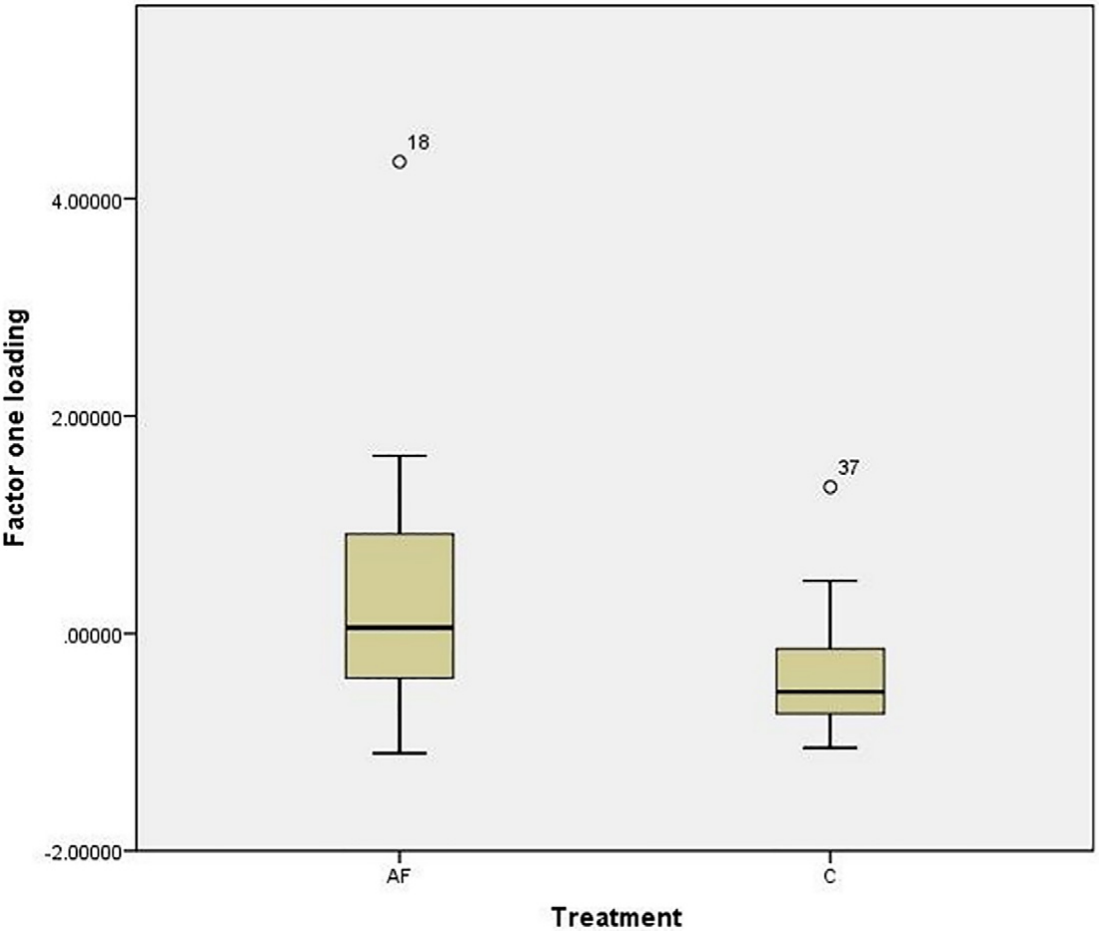
Boxplot (median ± interquartile range) of factor one “looking at and exploring object” loadings for AF group and C group.

Twenty cats in each group were included in the data analysis, three cats in each group being excluded because of suboptimal test conditions such as the cat being introduced to close to the object. Three factors were extracted during the factor analysis (see S4 File) that explained 79.9% of the variance. Factor one was named “looking at and exploring object”, factor two “anxiety/conflict like behaviour” and factor three “time spent near the object”. There was a significant difference between the groups for factor one “looking at and exploring object” (median loading ± IQR; AF group: 0.052 ± 1.53; C group: -0.54 ± 0.61; Mann Whitney U test; U = 107, Z = -2.516, p = 0.012), with AF cats exploring the object more than C cats (Fig 5). There were no significant differences between AF and C groups for factor two “anxiety/conflict like behaviour” and factor three “time spent near the object” (Mann Whitney U test; p > 0.05). Regarding individual behaviours of interest, two durations were selected to investigate the cats’ reaction to the test situation (“gaze towards the experimenter”, “gaze towards the door”), one for greeting behaviour (“tail up” duration) as well as “ear towards object” frequency. No significant differences were found for any of the behaviours (Mann Whitney U test, p > 0.05).

### Sudden noise test

Eighteen cats from the AF group and 20 cats of the C group were entered into the analysis, five cats in the AF group and three cats in the C group being excluded due to suboptimal test conditions, such as the cat stopping to eat before the noise was triggered. Two factors were extracted during the factor analysis (see S5 File), which explained 69.7% of the variance. Factor one was named “reaction to sudden noise”, factor two “non-reactivity” because “ear stationary” loaded positively while “non-feeding” duration loaded negatively on the factor. No significant differences were found between the groups for either factor (p > 0.05).

### Cognitive judgment bias test

Only five AF cats and nine C cats completed both the training and the testing phases, thus were included in the final model. Data were log10 transformed in order to achieve normality, as assessed by Shapiro-Wilk’s test (p > 0.05 for all locations and groups except AF group for near unrewarded location). Significant differences were found between response to the ambiguous locations, with latency to the near unrewarded location significantly higher (mean duration in seconds ± SE: AF cats 4.42 ± 1.95; C cats 2.89 ± 0.93) than the latencies for the near rewarded and the middle ambiguous locations (mean duration in seconds ± SE: NR AF cats 1.84 ± 0.27 C cats 1.25 ± 0.12; M AF cats 1.59 ± 0.2 C cats 1.4 ± 0.22; F = 7.072, p = 0.021). However, no significant differences were found between the AF and C groups, either for the number of trials taken to reach the criterion (mean number of trials ± SE; AF cats 15.5 ± 1.2 C cats 19.3 ± 1.44; average of 20 min to reach the criterion) or latencies to approach the ambiguous and training locations during testing (all p > 0.05).

### Owner questionnaire

**Fig 6:**
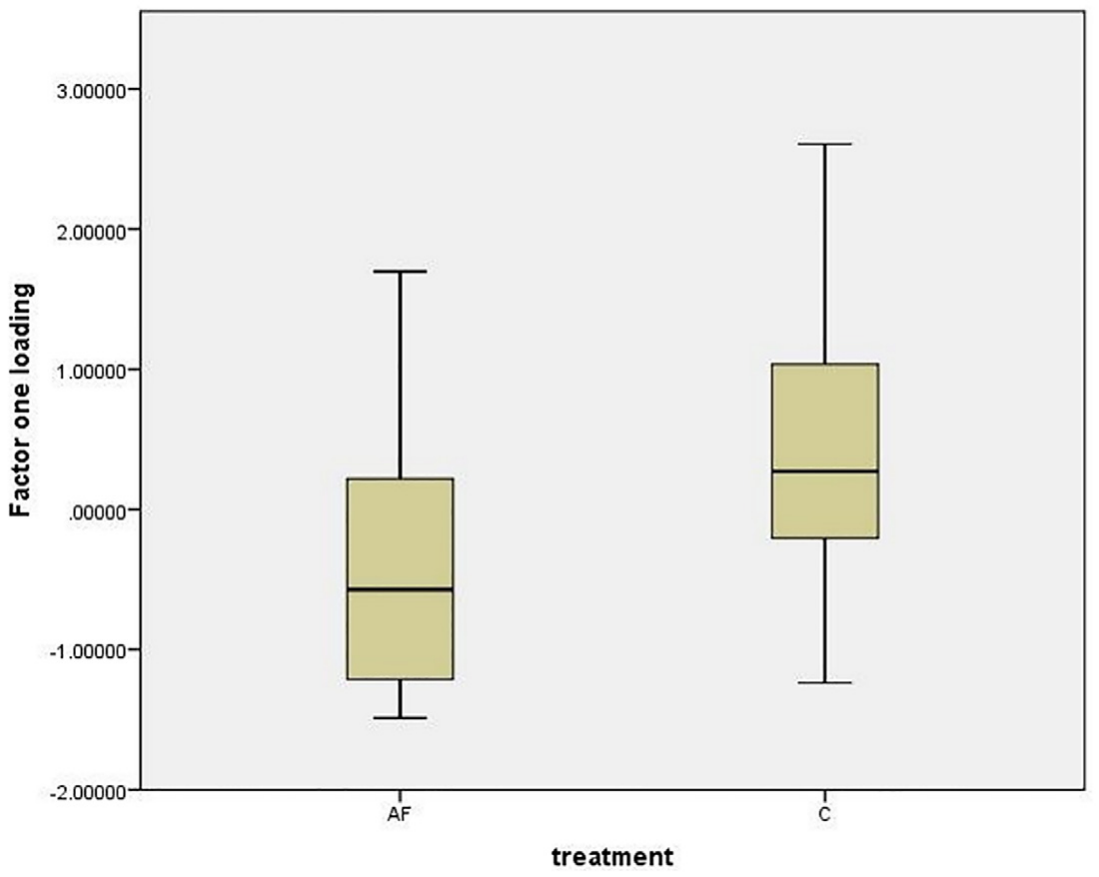
Boxplot (mean ± interquartile range) of “irritability” (factor one loading) for AF and C group.

**Fig 7:**
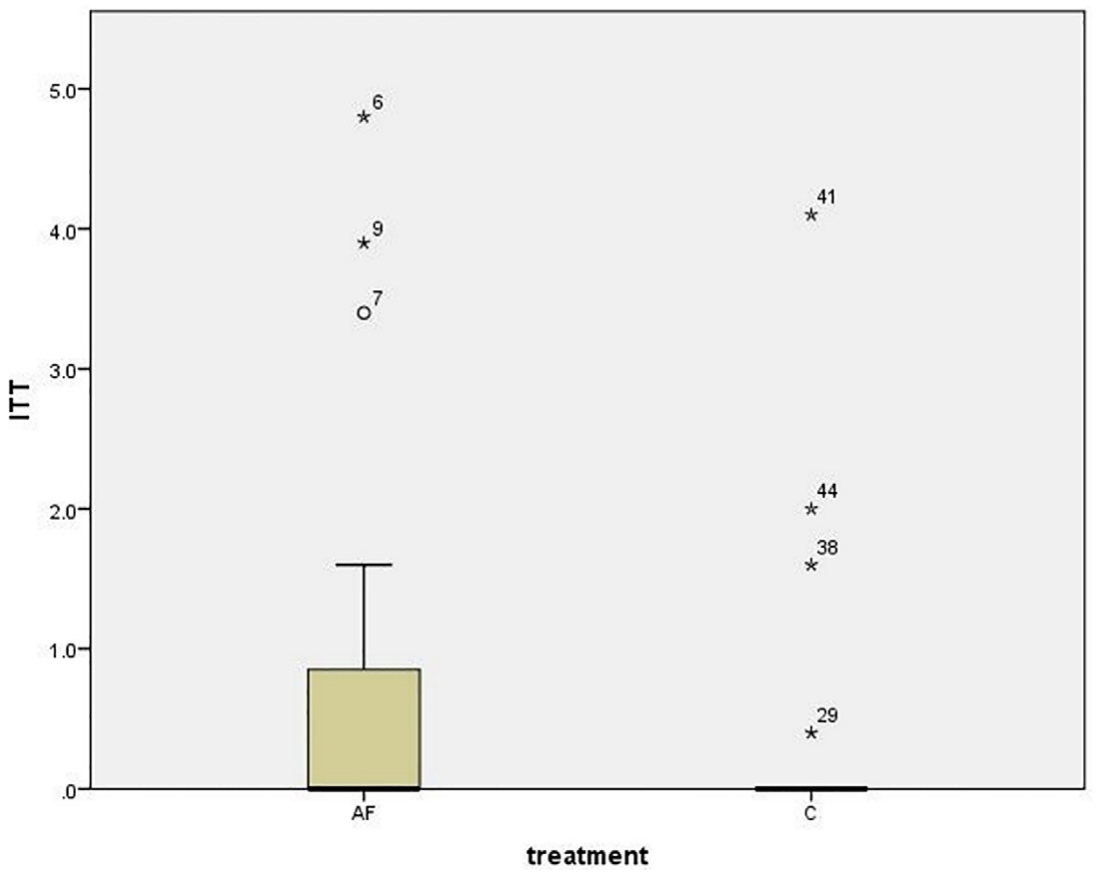
Boxplot (mean ± interquartile range) of “inappropriate toileting” frequency for AF and C group.

There were no statistically significant differences in the reports of owners regarding the time spent interacting with their cat, the time spent outdoors by the cat and their cat’s health. Most owners thought that their cat did not display any behaviour that could be considered unusual, abnormal or problematic and there was no significant difference between the groups in reporting such behaviours (p > 0.05). Regarding the 14 items relating to resources available to the cat in the home, there was a significant difference between AF and C groups for the item “litter tray”; 22/23 cats having access to a tray in the AF group compared to 14/23 in the C group; (Pearson’s chi square *X*^2^ = 8.178, p = 0.004). There was also a difference in access to the item “scratching post” (AF 21/23, C 14/23, Pearson’s chi square *X*^2^ = 5.855, p = 0.016). Regarding the behaviour observed by owners, three factors were extracted during the factor analysis (see S6 File). Factor one was named “irritability” because irritable behaviours load positively and “social interaction with cats” load negatively on that factor. Factor two was named “arousal” that can be in a indicating positive arousal “playing interaction with human” and “tail erected” (often during play according to owners) or stress with “long lasting hiding”. Factor three was the behaviour “hissing or growling”. C group owners thought that their cat showed more irritable behaviour than AF owners (median loading ± IQR; AF -0.57 ± 1.53; C 0.27 ± 1.43; Mann Whitney p = 0.022; see Fig 6). Whereas AF owners thought that their cats toileted inappropriately more often (median cm ± IQR AF 0.0±0.9; C 0.0±0 Mann Whitney p = 0.048; see Fig 7), although levels of inappropriate toileting were low for all cats. There were no significant differences between groups for the factor “arousal” or “hissing or growling” (both p > 0.05).

## Discussion

The aim of the study was to investigate the effects of long-term exposure to a virtual or invisible electronic containment system on cat behaviour and welfare by comparing cats that had already been exposed to the system for at least one year to those who had no experience of electronic containment. The putative effect on behaviour and welfare was assessed using a variety of approaches including an owner-based questionnaire, cognitive bias and behavioural tests. Relatively few cats completed the cognitive bias test, so firm conclusions based on a lack of difference are difficult to draw from this test; nonetheless it is clear that cats, compared to for example dogs [46], require considerably longer training and testing periods. There were no differences between the groups in the sudden noise test and few differences in the owner survey, unfamiliar person and novel object tests. AF cats interacted more with both the novel object and the unfamiliar person when she was active. This might indicate that the cats with experience of virtual fences were less cautious and/or that they were more motivated to interact with novelty. Enhanced interaction is generally interpreted as a sign of more positive affective state, suggesting better welfare for contained cats. This is supported by the observation that during phase one of unfamiliar person test, these cats displayed less “lip licking”, which has been associated with anxiety and stress [53, 54]. It might be expected that being alone with an unfamiliar person may be stressful for the cat, but this test was also the first of a series of disruptions which made up the series of tests, so the cat’s behavioural changes may have been a response to non-specific change rather than a specific response to the presence of the unfamiliar person. Some caution may be warranted in the interpretation of this result as it was the only significant result of five tests performed for this phase. Nonetheless, if there is an effect it is evidently in favour of the contained cats and this is supported by the results of the latter phases of this test.

Cats have been shown to interact more when a person is active [36]. In the unfamiliar person test, we also predicted that more stressed cats may interact less with the unfamiliar person during phases two and four (i.e. the phases when the unfamiliar person was active without then with the owner present but inactive). In line with the results of phase one; AF cats explored significantly more and interacted more with the stranger. It is possible that these responses are quite specific to unfamiliar people and may reflect differences in sociability or early experiences between the populations [35, 59], since there was no difference between the groups in the factors “confidence” or “positive behaviour” derived from the composition of their behaviour in the test and no difference outside of phase one in other behaviours that may be related to anxiety or conflict such as “lip licking”, “self-grooming” [54]. It is worth noting that both groups interacted with the stranger and showed greeting and positive affiliative behaviours, indicating that, overall, subjects were not avoidant and potentially in a positive affective state.

Regarding the novel object test, AF cats were again less neophobic, exploring the novel object more than C cats. There were no other significant differences between the groups in the display of anxiety or conflict like behaviours, nor in the time spent near the object, suggesting that the observed difference in exploration of the novel object might not be due to a wider difference in anxiety.

There was no significant difference between groups in the reaction to the sudden noise in any of the recorded measures, indicating that cats exposed to a beep in association with a potentially aversive electric stimulus (AF cats) were no more sensitive to a sudden or high pitched noise than control cats. This suggests there was no difference between the groups in vigilance or reactivity that might be associated with anxiety [60], nor evidence of widespread stimulus generalisation to such noises, which can occur when an individual is trained to associate a sound with an aversive consequence [61]. This might, in part, reflect the nature of the study being under field conditions, where external noise could not be completely suppressed, or the difference in location of the test compared to where the warning sound is normally heard [62]. It is worth noting that most of the cats’ responses were very mild, e.g. a subtle ear movement and even when there were signs of anxiety or conflict like behaviours, these rarely extended to the animal leaving the feeding area (less than 10% of the cats did leave the feeding area, and not immediately after the noise). This again indicates that the overall anxiety level of cats in both groups was very mild, compared to other animals tested in similar conditions [40, 42].

The cognitive judgment bias test was used to specifically assess the cats’ affective predisposition rather than their response to specific stimuli [42–44]. It has been used to infer both positive [61, 63–65] and negative [66, 67] affective states. No significant differences were found between the two groups. This is consistent with there being no difference in affective state but, it should be noted that the sample size, for this particular test was small with only 14 out of the 46 cats completing training and testing which indicates that the conclusions have to be drawn with caution. The high dropout rate, may reflect the requirement for cats to complete training within a day, since the only previously published study to apply cognitive bias tests to cats [45] achieved a much higher success rate (9/11 cats) but used between three and nine days of training for subjects. This latter study examined the feasibility of the protocol used and did not compare populations, but in general, the test does seem to be sensitive, achieving results with small populations in other species [68, 69].

Overall, the behavioural tests indicate that the cats who have experienced extended confinement using an invisible electronic containment system are generally less neophobic than cats not contained in this way and potentially free to roam outside of the property boundaries. This difference might be the result of greater exposure to uncontrolled aversive stimuli for the control cats, such as unfriendly neighbours, negative social interactions or traffic off-site or possibly a population selection bias, if the AF cats’ early experiences made them less neophobic [35]. For instance, AF cats could be less neophobic due to experiencing novelty within the confines of a more controlled environment. Nonetheless, there was no evidence of a significant difference between the two populations in either their affective predisposition or their tendency to generalise their response to the conditioned stimulus, which have been raised as a potential concern of these systems [10].

The owner questionnaire indicated that the cat owners recruited for this study have installed the containment electronic system, primarily because they feared for their cat’s safety–especially road traffic accidents. We studied 14 items relating to enrichment provision for the cat, and found significant differences only for two items at P<0.05. However, it might be argued that a threshold of P<0.003 may be more appropriate given the number of tests involved, in which case there were no significant differences between the two groups. Either way, it is clear most items were being provided for all the cats, indicating that the cats in this study were cared for to a high standard, regardless of group. AF owners provided more litter trays and more scratching posts. Although the estimated frequencies of “toileting inappropriately” was very low for both groups of cats, this was reported to occur more frequently amongst AF cats. This might explain the difference in the provision of litter trays, but the reason for the difference in the behaviour is not clear. We did not try to ascertain whether or not the behaviour related to marking or latrine related issues. The former has been associated with general anxiety in cats [70–72] whereas the latter relates to more local preferences and aversions with the latrine options provided [73–76]. There were no indications from either the behaviour tests or survey to indicate that the cats in the AF group were more anxious or that the increased prevalence of problem might be related to the emotional consequences of using the electronic containment system, even in the AF multi-cat households. Indeed, it is worth noting that the only owner reported difference in behaviour possibly related to emotionality indicated that the C cats, not the AF cats, showed significantly more irritable behaviour.

The owners were asked to report on their cats’ behaviour over the previous year and the lack of differences between the two populations may reflect little underlying difference in their overall state or a lack of sensitivity of the metric used. It might be that owners do not attend to subtle behaviours, such as “lip licking” “skin twitching” or “head shaking” or appreciate their potential significance. Although owners have been shown to be able to classify the vocalisation of their own cat, the rate of classification is not high on average [50], perhaps reflecting a wider lack of attention to potential feline communicative signals. Nonetheless, owner’s perceptions can be a useful tool for behaviour analysis in some circumstances [29].

In conclusion, we recorded a range of different measures, from the cat itself as well as indirect observations obtained via the cat’s owner, in order to investigate the potential long-term impact of an invisible electronic containment system on cat behaviour and welfare. Taken together, the findings do not suggest that long-term (at least 12 months) exposure to the system had a significant negative impact on the behaviour and welfare of contained cats. Indeed cats subject to electronic confinement appear to be less neophobic than unrestrained cats. This might relate to the containment increasing predictability in the environment and deserves further investigation. Owner report and the behavioural and judgment bias tests found no evidence of a difference in the general anxiety of the two populations of cats studied. Further investigation could usefully address the short-term impact of installing containment systems on the behaviour and welfare of cats, and the potential influence of previous roaming experience, but regardless of this, current evidence, reported here, indicates that these systems have the potential to reduce roaming by cats and its consequential risks without causing long term distress. Future research might usefully assess the welfare impact of other approaches to restricting cats from roaming, such as keeping the cat indoors, since this too has the potential to give rise to welfare concerns [77, 78].

## Supporting Information

S1 File (PDF)

S2 File (PDF)

S3 File (PDF)

S4 File (PDF)

S5 File (PDF)

S6 File (PDF)

S7 File (XLSX)
