# Supplementary material for: Effects of Long-Term Exposure to an Electronic Containment System on the Behaviour and Welfare of Domestic Cats
Source: PLoS One. 2016 Sep 7;11(9):e0162073. doi: 10.1371/journal.pone.0162073 (PMC5014424; doi:10.1371/journal.pone.0162073)
Supplement: S1 File — (PDF) [file pone.0162073.s001.pdf]

UNIVERSITY OF
LINCOLN

Dear Sir/Madam,

Thank you very much for volunteering to take part in our study. This study is being undertaken by researchers at the University of Lincoln to assess the potential effect of containment systems on cat welfare. We would like to perform some simple behavioural tests with your cat to see how he/she responds in different contexts, for example when meeting a new person. These tests, in conjunction with talking to you about your observations of your cat's behaviour (see below), will help us to assess the welfare of your cat. For this reason, your input and the knowledge you have of your cat is very valuable to our study.

This questionnaire should be filled in by the adult who spends most time with the cat, and, when answering the following questionnaire about the behaviour of your cat, please take your time to answer as accurately as possible. Please include all your observations relating to a given question, even if you think that they might not be important – we simply want to try to understand your cat's behaviour.

I will be available to answer any questions that you might have whilst you complete the questionnaire, and can provide you with examples (via photos and video clips) of the key cat behaviours that we are interested in, so please do not hesitate if there is anything that you wish to ask.

We thank you again for your help,

Yours faithfully,

Dr Naima Kasbaoui

Other research team members: Prof D. Mills, Prof J Cooper, Dr O. Burman, Dr Marta Gil

1. **Please give the name of your cat:**

…….

2. **Is your cat male or female?**

☐Male

☐Female

3. **Is your cat neutered?**

☐Yes

☐No

4. **If Yes, at what age was it neutered? (if not sure, please indicate this)**

…….

5. **Has your cat learned to do any behaviours for a reward? For example…..sitting on command for a treat?**

☐Yes

☐No

6. **Before the installation of the electronic containment fence, did you do anything to contain your cat? (like having high fences, supervised access outdoor, cat-proof fences)**

☐I did not do anything to contain my cat

☐I kept my cat indoors

☐My cat had a supervised access outdoors (e.g. I put him/her on a leash or I watch him/her)

☐I have high fences

☐I have specific « cat-proof » fencing (such as those shown in the images below) :

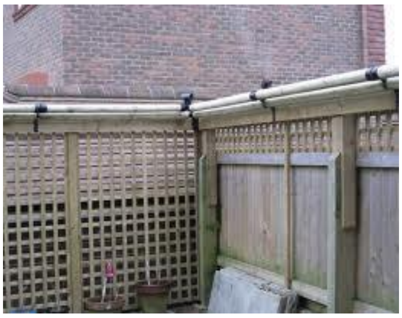

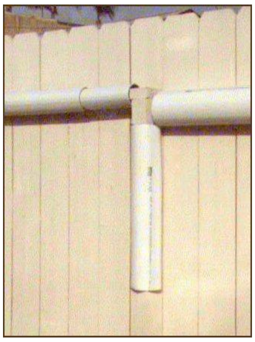

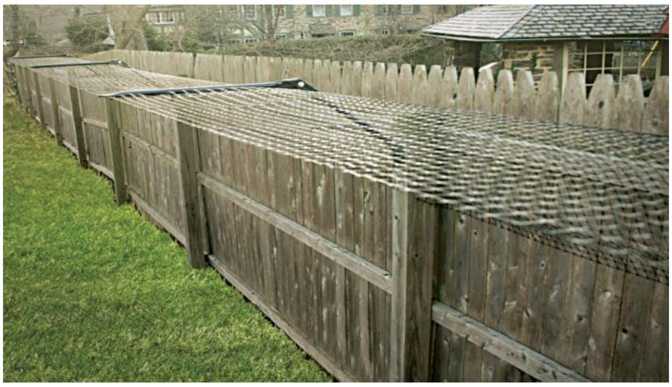

cat secure®, purrfect fence®

☐I have another specific containment system, please specify :
…….

7. **Could you please state the make and model of the electronic containment system you chose to have installed, and when you had it installed?**

Make: ……….

Model: ……….

Date of installation: ………………………………………………………………………………………

Size of the contained area: ……………………………………………………………………………….

8. **Before the electronic containment system installation, what was the level of access your cat had outside? (please tick one of the following, and provide more detail where indicated)**

☐ All day

☐ All night

☐ A few hours a day, please state how many: ……………………………………………………………….

☐A few hours at night, please state how many: …………………………………………………………

☐ 24/7 access outside (e.g. through a cat flap or living outside)

9. **What was your rationale for installing an electronic containment system?**

……
……
……
……
…….

10. **How many times, if any, in the last week do you think your cat has received an auditory warning from its collar?**

……
……
……
……
…….

11. **How many times, if any, in the last week do you think your cat has received an electric stimulation from its collar?**

……
……
……
……
…….

12. **Are there any special places in the contained area you think your cat likes to go?**

……
……
……
……
………

13. **What type of food does your cat eat and at what time in the day? Please state the type of food (dry or wet food, if you know it the brand, and the quantity per meal). If the cat is fed as much as he/she wants, please state the quantity you give him/her per day.**

Food type and quantity: ……………………………………………………………………………………………

Time fed: ……..

Food type and quantity: ……………………………………………………………………………………………

Time fed: ……..

Food type and quantity: ……………………………………………………………………………………………

Time fed: ……..

Food type and quantity: ……………………………………………………………………………………………

Time fed: ……..

14. **How much time do you and other members of the family spend interacting with your cat (playing, stroking him or her, sitting together etc) on a typical 24 hours?**

☐ Less than 1 hour

☐ 1-2 hours

☐ 2-5 hours

☐ More than 5 hours

15. **How much time do you think your cat spends outside over an average 24 hours?**

☐ Less than 1 hour

☐ 1-2 hours

☐ 2-5 hours

☐ More than 5 hours

16. Which of the following do you provide for your cat inside the home? (please tick all that apply)

☐ Opportunity to exercise beyond normal walking in the house (e.g. play, movable feeding devices)

☐ Access to a spot for sun bathing

☐ Water

☐ Toys

☐ Access to fresh air (e.g. partially opened window)

☐ Food

☐ Litter tray

☐ Specific cat sleeping area

☐ Scratching post

☐ Companionship from other animals

☐ Companionship from humans

☐ Vantage points

☐ Places to hide

☐ Windows to watch outside

☐ Other specific provisions to help keep your cat happy, please give details

……

……

17. Would you say that your cat is in good health?

☐ Yes

☐ Not sure

☐ No. Please state the type of problems your cat has:

……

……

18. Would you say that your cat is stressed?

☐ No

☐ Not sure

☐ Yes. Please state the type of problems your cat has:

……

……

19. **Does your cat show any behaviours that you consider to be unusual, abnormal or problematic?**

☐ No

☐ Not sure

☐ Yes . Please give brief details here:

………

………

20. **If you had to describe your cat, do you think your cat is (<u>please circle the correct answer)</u>**

Very motivated by food / motivated by food / not very motivated by food / not motivated by food at all.

For the next questions, please put a cross on the line to rate your cat's behaviour and appetite.

21. **How would you rate your cat's anxiousness?**

Not anxious at all ———————————————— Very anxious

22. **Would you say your cat is outgoing/confident?**

Not outgoing at all ———————————————— Very outgoing

23. **How would you rate your cat's appetite?**

Poor ———————————————— Good

24. When a NEW OBJECT is introduced into the house, how does your cat react?

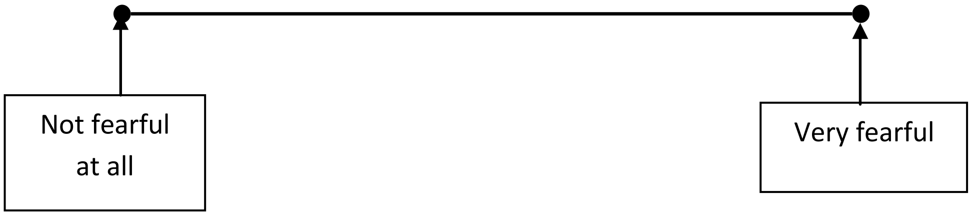

25. When a NEW PERSON comes into your house, how does your cat react?

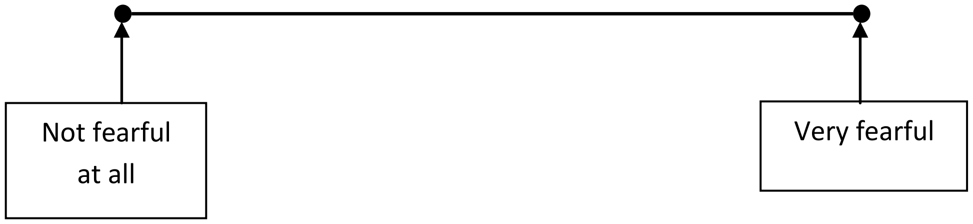

26. Overall, how well do you think your cat copes with CHANGES (e.g. moving furniture, having some building/maintenance work done at your home, moving house, new people coming round etc.,) Please put a cross on the line below to indicate your response. If your cat has specific events, that it dislikes, please indicate these afterwards

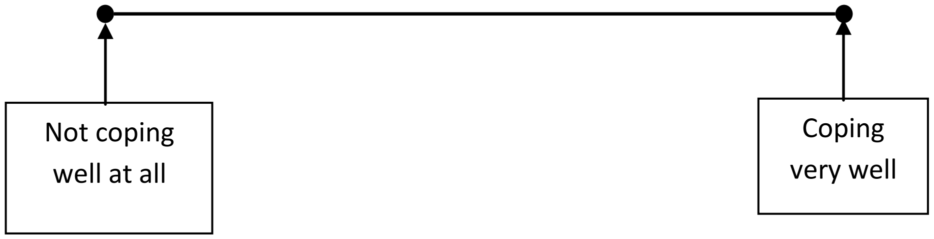

Specific event that your cat dislikes:

………
………
………
………
………

27. Have there been any significant changes, (including, but not limited to, those examples listed above in question 26) to the household in the last six months?

☐No

☐Yes (please state below any changes and also the approximate date at which they happened.)

………
………
………
……

28. Please state the household composition

Number of adults: ………

Number of children and their age:
………
………

29. How would you rate the quietness of your household? (please put a cross on the line to mark the rate)

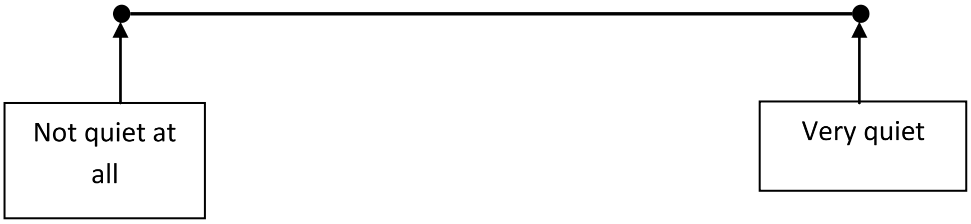

YOUR CAT'S BEHAVIOUR <u>IN THE LAST WEEK</u>

Given below is a list of cat behaviours. For each behaviour, please record *on the first line* the *typical frequency* that your cat shows the behaviour.

On *the second line* please record, in the same way, the frequency of the same behaviour but just describing *your cat's behaviour during the last week*.

For example, if your cat typically shows a particular behaviour once a week or less, then this is very infrequent and so you would put a mark on the line closer to the left hand end. If, however, in the last week your cat has been showing this same behaviour several times a day, then…

☐Long lasting hiding (e.g. in boxes or hiding places, behind the sofa, under the bed)

Typical frequency

Never ●————————● Very frequent

Last week frequency

Never ●————————● Very frequent

☐Hissing or growling in any context (e.g. at you, others cats in the home, or a neighbour's cat)

Typical frequency

Never ●————————● Very frequent

Last week frequency

Never ●————————● Very frequent

☐Scratching objects, including its own scratch post

Typical frequency

Never Very frequent

Last week frequency

Never Very frequent

☐Scratching or biting people (owners or visitors)

Typical frequency

Never Very frequent

Last week frequency

Never Very frequent

☐Fighting with other cats (either with your own, if you own more than one cat, or other people's cats)

Typical frequency

Never Very frequent

Last week frequency

Never Very frequent

☐Lip licking or exaggerated swallowing without any food involved

Typical frequency

Never Very frequent

Last week frequency

Never Very frequent

☐Short sharp and rapid self-groom/ licking

Typical frequency

Never Very frequent

Last week frequency

Never Very frequent

☐Head shaking (refer to example)

Typical frequency

Never Very frequent

Last week frequency

Never Very frequent

☐Skin twitching or rippling (refer to example)

Typical frequency

Never Very frequent

Last week frequency

Never Very frequent

☐ Tail erected or body hair erected (refer to example)

Typical frequency

Never Very frequent

Last week frequency

Never Very frequent

☐Inappropriate toileting: spraying, defecating or urinating in the house

Typical frequency

Never Very frequent

Last week frequency

Never Very frequent

☐Social interactions with you (rubbing on your leg, hands, coming for a stroke, purring next to you or when stroked, sitting or lying next to you or on your knees, sleeping with you..)

Typical frequency

Never Very frequent

Last week frequency

Never Very frequent

☐Social interaction with other cats (rubbing on each other, grooming each other, sleeping in very close proximity, walking alongside one another)

Typical frequency

Never Very frequent

Last week frequency

Never Very frequent

☐Playing interaction with you or with a toy

Typical frequency

Never Very frequent

Last week frequency

Never Very frequent

☐Interacting with toys (on his own), feeding devices and/or any forms of enrichment

Typical frequency

Never Very frequent

Last week frequency

Never Very frequent
