# Supplementary material for: Effects of Long-Term Exposure to an Electronic Containment System on the Behaviour and Welfare of Domestic Cats
Source: PLoS One. 2016 Sep 7;11(9):e0162073. doi: 10.1371/journal.pone.0162073 (PMC5014424; doi:10.1371/journal.pone.0162073)
Supplement: S2 File — (PDF) [file pone.0162073.s002.pdf]

Ethogram unfamiliar person test

Category	Behaviour	Description
Location	Zone1 o s	Cat's head and at least one front leg is in contact or at less than half cat length from the Stranger (s) or owner (o)
	Zone 2 o s	Cat's head and at least one front leg is between half a cat's length and one metre from the stranger (s) or the owner (o)
	Zone 3	Cat's head and at least one front leg is at more than one metre from any of the human present
	Zone 4	Cat's head and at least one front leg is outside cameras fields
Posture and locomotion	Stand	Cat not mobile, at least three paws on floor limbs extended
	Crouch	Cat not mobile at least three paws on floor limbs bent to a degree, belly can be touching the ground or not.
	Sit	Cat's hindquarters and at least three paws are on the floor. Front legs are extended.
	Jump	Cat leaps from one point to the other, either vertically or horizontally.
	Lay down	Cat's body is on the floor in a horizontal position, including on its side, belly or curled in a circular formation.
	Roll	While on the floor, cat rotates its body from one side to another, sometimes staying on its back not mobile.
	Roll with physical contact	While on the floor, cat rotates its body from one side to another, sometimes staying on its back not mobile then any part of its body stays in contact with owner or stranger
	Freeze	Cat's body is completely still and tense for at least two seconds
	Walk	Forward locomotion at a slow gait

	Run	Forward locomotion at a faster gait than walking. Include trotting and galloping
	Other	Cat has a posture or locomotion that is none of the above
	Not visible	Cat is not visible
Gaze	Gaze towards the stranger	Cat looks towards the stranger
	Gaze towards the owner	Cat looks towards the owner
	Gaze elsewhere	Cat looks anywhere but not towards any human present
	Gaze not visible	Cannot see where the gaze is directed
Cat human interaction	Sniffing o s	Cat is sniffing the owner or the stranger
	Rubbing o s	Cat is rubbing its head or any part of its body on the owner or the stranger
	Contact o s	Cat’s body or head is touching the owner or the stranger
	Biting o s	Cat is biting the owner or the stranger
	Initiate interaction o s	By its behaviour cat asked to be stroked by the owner or the stranger
	Headbutt event o s	Cat is giving a headbutt to the owner or the stranger
	Licking o s	Cat is licking the owner or the stranger
	Kneading	Cat pushes forepaw into the floor or cushion or blanket or couch in a rhythmic kneading motion
	Other interaction	Cat interacts with owner/stranger in a way non listed
Actions	Self-grooming	Cat is licking itself on any part of its body
	Short sharp rapid groom	Cat licks itself but rapidly and only two or three times
	Lip-licking	Cat is licking its lips or swallowing when food is not involved. Or cat’s tongue is in and out of the mouth in a rapid movement.
	Head shaking	Cat rotates its head very quickly from side to side

	Skin twitching	Cat skins twitches or ripples.
	Other	Cat doing anything else
Vocalisation	Miaowing	Cat is making calling noise
	Purring	Low, continuous rhythmical tone produced during respiration while the cat's mouth is closed. Creates a murmuring sound.
	Hissing	Cat is making a drawn-out low intensity hissing sound produced by rapid expulsion of air from the cat's mouth.
	No vocalisation	Cat is not vocalising

Ethogram novel object test

**Behaviour group**	**Behaviour**	**Description**
Location	Near Object	Cat's head and at least one front leg is within 20 centimetres of the object
	Around Object	Cat's head and at least one front leg is between 20 centimetre and one metre of the object
	Away from Object	Cat's head and at least one front leg is farther than one metre of the object
Posture and locomotion	Stand	Cat not mobile, at least three paws on floor limbs extended
	Crouch	Cat not mobile at least three paws on floor limbs bent to a degree, belly can be touching the ground or not.
	Sit	Cat's hindquarters and at least three paws are on the floor. Front legs are extended.
	Jump	Cat leaps from one point to the other, either vertically or horizontally.
	Lay down	Cat's body is on the floor in a horizontal position, including on its side, belly or curled in a circular formation.
	Roll	While on the floor, cat rotates its body from one side to another, sometimes staying on

		its back not mobile.
	Freeze	Cat's body is completely still and tense for at least two seconds
	Walk	Forward locomotion at a slow gait
	Run	Forward locomotion at a faster gait than walking. Include trotting and galloping
	Other	Cat has a posture or locomotion that is none of the above
Gaze	Gaze towards the object	Cat looks towards the object
	Gaze towards the experimenter	Cat looks at the experimenter
	Gaze elsewhere	Cat looks anywhere but not towards the object
	Gaze not visible	Cannot see where the gaze is directed
Tail	Tail up	Tail is held in an upright position
	Tail anywhere	Tail is held in any position or has any movement but not upright
	Tail out of sight	More than 10cm from the tip of the cat's tail is not in view
Contact with object	sniffing	Cat smells object by inhaling air through nose
	rubbing	Cat is rubbing its head or any part of its body on the object
	touching	Any part of cat body or head is in direct contact with the object, without rubbing
	chewing	Cat grinds the object with its teeth
	Biting	Cat snaps teeth at and is successful in biting object
	Head butt	Cat gives a head but to the object

	Other type of contact	Cat is in contact with object in a way that is none of the above
Actions	Self-grooming	Cat is licking itself on any part of its body
	Short sharp rapid groom	Cat licks itself but rapidly and only two or three times
	Head shaking	Cat rotates its head very quickly from side to side
	Yawning	Cat opens its mouth widely while inhaling, then closes mouth while exhaling deeply.
	Lip-licking	Cat is licking its lips or swallowing when food is not involved. Or cat's tongue is in and out of the mouth in a rapid movement.
	Skin twitching	Cat skins twitches or ripples.
	Kneading	Cat pushes forepaw into the floor or cushion or blanket or couch in a rhythmic kneading motion
Vocalisation	Miaowing	Cat is making calling noise
	Purring	Low, continuous rhythmical tone produced during respiration while the cat's mouth is closed. Creates a murmuring sound.
	Hissing	Cat is making a drawn-out low intensity hissing sound produced by rapid expulsion of air from the cat's mouth.
	No vocalisation	Cat is not vocalising
Ear Movement	Ears anywhere	Ears not moving towards object
	Ears towards object	Ears moving towards object

Ethogram sudden noise test

Category	Behaviour	Description
Location	At bowl	Cat is within one head length of the bowl **and could move solely it's head to allow it to reach the bowl to feed**
	Near bowl	Cat is between one head length and one body length of the bowl
	Far from bowl	Cat is more than one body length from the bowl
Feeding status	Feeding	The cat is selecting food from its bowl and/or chewing
	Non-feeding	The cat is not selecting food from its bowl or chewing
Body position	Sit	Cat’s hindquarters and at least three paws are on the floor. Front legs are extended.
	Stand	Cat not mobile, at least three paws on floor limbs extended
	Crouch	Cat not mobile at least three paws on floor limbs bent to a degree, belly can be touching the ground or not.
	Freeze	Cat's body is tense and completely still for at least two seconds
	Other	Cat is exhibiting any body position not mentioned
	Body out of sight	Not enough of the cat's body is in view to determine its position at above chance level
Head position	Head towards speaker	**Cat's head is to one side of its centre line in the direction of the speakers. OR**<br><br>**Cat's head is at any point between slightly turned towards and fully facing the speakers.**

	Head anywhere, but not towards speaker	Cat's head is at any position apart from towards the speaker
Ear position	Ears towards speaker	Cat's ears move independently or together towards the speaker
	Ears not towards speaker	Cat's ears move independently or together in any direction apart from towards the speaker
	Ears stationary	Cat's ears are still
	Ears out of sight	Both of the cat's ears are not in view
Ear stationary positions	Ears forwards	Cat's ears, specifically the tips, are turned to the front of the head
	Ears backwards	Cat's ears, specifically the tips, are turned to the back of the head
	Ears sideways	Cat's ears, specifically the tips, are lateral on the head
	Other	Cat is exhibiting any stationary ear position not mentioned
Tail position	Tail flick	Cat moves 5cm or less of the tip of it's tail
	Tail sway	Cat moves its entire tail from side to side
	Tail stationary	There is no movement of any part of the tail
	Other	Cat is exhibiting any tail movement not mentioned
	Tail out of sight	More than 10cm from the tip of the cat's tail is not in view
Movement direction	Leaving feeding area	Cat is moving away from the feeding area
	Returning to	Cat is moving towards the feeding area

	feeding area	
Locomotion	Walk	Forward locomotion at a slow gait
	Run	Forward locomotion at a faster gait than walking. Include trotting and galloping
Actions	Self-grooming	Cat is licking itself on any part of its body
	Lip-licking	Cat is licking its lips or swallowing when food is not involved. Or cat’s tongue is in and out of the mouth in a rapid movement.
	Other	Cat is exhibiting any action not mentioned
Vocalisation	Miaowing	Cat is making calling noise
	Other	Cat is exhibiting any vocalisation not mentioned
