# Supplementary material for: Effects of Long-Term Exposure to an Electronic Containment System on the Behaviour and Welfare of Domestic Cats
Source: PLoS One. 2016 Sep 7;11(9):e0162073. doi: 10.1371/journal.pone.0162073 (PMC5014424; doi:10.1371/journal.pone.0162073)
Supplement: S3 File — (PDF) [file pone.0162073.s003.pdf]

Factor analysis supplementary information

Unfamiliar person test

Phase 2: unfamiliar person alone with cat, active

The FA yielded a KMO measure of 0.695. Bartlett's test of sphericity (chi square (15)=51.881, $p<0.0005$) indicated that the data were suitable for a FA. Two factors were extracted during the analysis, which explained 64.66% of the variance. Factor one was labelled as "Looking at and exploring the stranger", and factor two "Confidence" as the greeting behaviour load positively on the factor while the anxiety/conflict like behaviours load negatively on this factor.

Table 1: Behaviour variables loadings on the two factors of interest extracted.

Behaviour	factor one: looking at and exploring the stranger	factor two: confidence
SSD	0.883	
SSF	0.868	
GTSD	0.799	
TUD		0.815
LLF		-0.695
SGD		-0.617

SSD=sniffing stranger duration SSF=sniffing stranger frequency GTSD=gaze towards stranger duration TUD=tail up duration LLF=lip licking frequency SGD=self-grooming duration

Phase 3: owner and unfamiliar person present, both passive

The FA yielded a KMO measure of 0.637. Bartlett's test of sphericity (chi square (15)=35.645, $p=0.005$) indicated that the data was suitable for a FA. Two factors were extracted during the analysis, which explained 60.37% of the variance. Factor one was named "looking at owner and greeting behaviour", and factor two "looking at stranger and positive behaviour" as "gaze towards stranger" loaded positively on this factor while the anxiety/conflict like behaviours loaded negatively.

Table 2: Behaviour variables loadings on the two factors of interest extracted.

Behaviour	factor one: looking at owner and greeting behaviour	factor two: looking at stranger and positive behaviour
GTOF	0.818	
GTOD	0.813	
TUD	0.61	
GTSD		0.694
MF		-0.601
LLF		-0.529

GTOF=gaze towards owner frequency GTOD=gaze towards owner duration GTSD=gaze towards stranger duration MF=meowing frequency TUD=tail up duration LLF=lip licking frequency

Phase four: owner passive, unfamiliar person active

The FA yielded a KMO measure of 0.691. Bartlett's test of sphericity (chi square (28)=81.913, $p<0.0005$) indicated that the data were suitable for a PCA. Two factors were extracted during the analysis, which explained 60.81% of the variance. Factor one was named "interaction with stranger", and factor two "gazes and positive behaviour" as the greeting behaviour loaded positively on this factor while the anxiety/conflict like behaviours loaded negatively.

Table 3: Behaviour variables loadings on the two factors of interest extracted.

Behaviour	factor one: interaction with stranger	factor two: gazes and positive behaviour
SSF	0.913	
IISF	0.763	
RSF	0.736	
SSD	0.641	
SGF		-0.797
TUD		0.722
GTSF		0.655
GTOF		0.486

SSF=sniffing stranger frequency IISF=initiate interaction with stranger frequency RSF=rubbing on stranger frequency SSD=sniffing stranger duration SGF=self-grooming frequency TUD=tail up duration GTSF=gaze towards stranger frequency GTOF=gaze towards owner frequency
