# Supplementary material for: Effects of Long-Term Exposure to an Electronic Containment System on the Behaviour and Welfare of Domestic Cats
Source: PLoS One. 2016 Sep 7;11(9):e0162073. doi: 10.1371/journal.pone.0162073 (PMC5014424; doi:10.1371/journal.pone.0162073)
Supplement: S4 File — (PDF) [file pone.0162073.s004.pdf]

Factor analysis supplementary information

Novel object test

The FA yielded a KMO measure of 0.657. Bartlett's test of sphericity (chi square (28)=179.742, $p<0.0005$) indicated that the data were suitable for a PCA. Three factors were extracted during the analysis that explained 79.91% of the variance. Factor one was named "looking at and exploring object", factor two "anxiety/conflict like behaviour" and factor three "time spent near the object".

Table 1: Behaviour variables loadings on the three factors of interest extracted.

Behaviour	factor one: looking at and exploring object	factor two: anxiety/conflict like behaviour	factor three: time spent near the object
SOD	0.93		
GTOD	0.908		
SOF	0.886		
SGF		0.884	
SGD		0.838	
LLF		0.724	
NOF			0.893
NOD			0.709

SOD=sniffing object duration GTOD=gaze towards the object duration SOF=sniffing object frequency SGF=self-grooming frequency SGD=self-grooming duration LLF=lip licking frequency NOF=near object frequency NOD=near object duration
