# Supplementary material for: Effects of Long-Term Exposure to an Electronic Containment System on the Behaviour and Welfare of Domestic Cats
Source: PLoS One. 2016 Sep 7;11(9):e0162073. doi: 10.1371/journal.pone.0162073 (PMC5014424; doi:10.1371/journal.pone.0162073)
Supplement: S5 File — (PDF) [file pone.0162073.s005.pdf]

Sudden noise test

The FA yielded a KMO measure of 0.701. Bartlett’s test of sphericity (chi square (36)=226.523, $p<0.0005$) indicated that the data were suitable for a PCA. Two factors were extracted during the analysis, which explained 69.67% of the variance. Factor one was named “reaction to the sudden noise”, factor two “non reaction” because “ear stationary” load positively while “non feeding” duration load negatively on the factor.

Table 1: Behaviour variables loadings on the two factors of interest extracted.

Behaviour	Factor one: reaction to the sudden noise	Factor two: non reaction
HTSF	0.88	
HTSD	0.852	
NFF	0.813	
ESF	0.774	
HTSL	-0.771	
LLF	0.742	
NFL	-0.68	
ESD		0.831
NFD	0.576	-0.597

HTSF=head towards speakers frequency HTSD=head towards speakers duration NFF=non feeding frequency ESF=ears stationary frequency HTSL=head towards speakers latency LLF=lip licking frequency NFL=non feeding latency ESD=ears stationary duration NFD=non feeding duration
