# Supplementary material for: Effects of Long-Term Exposure to an Electronic Containment System on the Behaviour and Welfare of Domestic Cats
Source: PLoS One. 2016 Sep 7;11(9):e0162073. doi: 10.1371/journal.pone.0162073 (PMC5014424; doi:10.1371/journal.pone.0162073)
Supplement: S6 File — (PDF) [file pone.0162073.s006.pdf]

Factor analysis supplementary information

Owner questionnaire

The FA yielded a KMO measure of 0.624. Bartlett's test of sphericity (chi square (21)=39.424, p=0.009) indicated that the data is suitable for a PCA. Factor one was named "irritability" because irritability behaviours load positively and "social interaction with cats" load negatively on that factor. Factor two was named "arousal" that can be in a indicating positive arousal "playing interaction with human" and "tail erected" (often during play according to owners) or stress with "long lasting hiding". Factor three is the behaviour "hissing or growling"

Table 1: Behaviour variables loadings on the three factors of interest extracted.

Behaviour	factor one "irritability"	factor two "arousal"	factor three "hissing or growling"
SICT	-0.808		
STT	0.719		
HST	0.487	0.484	
PIHT		0.85	
TET		0.635	
LLHT		0.525	
HISST			0.881

SICT=social interaction with other cat typical frequency STT=skin twitching typical frequency HST=head shaking typical frequency PIHT=playing interaction with human typical frequency TET=tail erected typical frequency LLHT=long lasting hiding typical frequency HISST=hissing or growling typical frequency
